# Management and Prognosis of Patients With Acute Pericarditis in the Emergency Department: A Retrospective, Single‐Centre Study

**DOI:** 10.1111/eci.70206

**Published:** 2026-04-24

**Authors:** Lisa Caldera, Chiara Lovati, Alessandra Vecchié, Walter Ageno, Marco Paolo Donadini, Francesco Dentali, Flavio Tangianu, Aldo Bonaventura

**Affiliations:** ^1^ Emergency Medicine Residency Program University of Milan‐Bicocca Monza Italy; ^2^ Pediatrics Residency Program University of Milan Milan Italy; ^3^ Medical Center, S.C. Medicina Generale 1, Department of Internal Medicine Ospedale di Circolo and Fondazione Macchi, ASST Sette Laghi Varese Italy; ^4^ Department of Medicine University of Padua Padua Italy; ^5^ Department of Medicine and Surgery University of Insubria Varese Italy

**Keywords:** acute pericarditis, chest pain, colchicine, emergency department, NSAIDs, prognosis, recurrent pericarditis

## Abstract

**Background:**

Despite being a common reason for Emergency Department (ED) admission, information about the management of acute pericarditis is limited in this setting.

**Methods:**

In this retrospective study conducted at the ED of Ospedale di Circolo in Varese (Italy) from 2019 to 2023, patients with acute pericarditis were included. The primary endpoint was the occurrence of the 12‐month composite outcome (treatment failure, recurrent pericarditis, cardiac tamponade, constrictive pericarditis or death).

**Results:**

One‐hundred and sixty‐nine patients were included (median age 54 years, 65.1% males). Chest pain was the main symptom (96.4%). On admission, aspirin was more frequently given over non‐steroidal anti‐inflammatory drugs (NSAIDs), and colchicine was prescribed in 40% of patients. At discharge, more patients were prescribed ibuprofen, and colchicine prescription significantly increased to 71%. Drug doses were compliant with guidelines in a limited number of patients at admission and increased at discharge. The composite outcome occurred in 20.1% of patients (*n* = 34), mainly driven by recurrences (*n* = 18) and treatment failure. Patients with a complicated course were older, of female sex, with a larger proportion of comorbidities and higher CRP levels. Diabetes (HR 3.9, 95% CI 1.7–9.1), COPD (HR 6.2, 95% CI 2.3–17.1), recent percutaneous cardiac procedures (HR 6.5, 95% CI 2.1–19.6), and recent SARS‐CoV‐2 vaccination (HR 3.0, 95% CI 1.1–8.2) were independent risk factors for the composite outcome.

**Conclusion:**

A significant proportion of patients with acute pericarditis experience long‐term complications. Sub‐optimal adherence to guideline‐recommended doses of anti‐inflammatory drugs was commonly observed, suggesting an area for improvement in the management of these patients.

## Graphical abstract

In this retrospective, single‐centre study conducted in patients with acute pericarditis referred to the Emergency Department, a significant proportion of patients experienced long‐term complications, especially recurrences. Drug doses were compliant with guidelines in a limited number of patients at admission and increased at discharge. Diabetes, COPD, recent percutaneous cardiac procedures, and recent SARS‐CoV‐2 vaccination were independent risk factors for the composite outcome. In addition, adherence to guideline recommendations for the doses of anti‐inflammatory drugs was sub‐optimal.

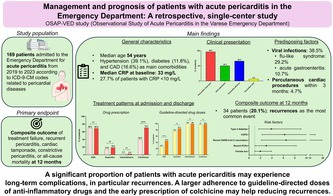

## 1 Introduction

Acute pericarditis is a clinical syndrome characterized by the inflammation of the pericardial sac that may cause concurrent inflammation of the myocardium—myopericarditis [1]. A not negligible proportion of patients experiences complications, especially recurrences [2, 3].

Patients with acute pericarditis usually present with chest pain and are referred to the Emergency Department (ED) to obtain a first medical evaluation; however, information about clinical presentation and management in this setting is scant [4, 5, 6, 7, 8, 9]. In a study published in 2022, the incidence of pericarditis and myopericarditis among patients with acute chest pain who presented to seven emergency departments in Switzerland was 1.9% and 1.1%, respectively [8]. The majority of these patients were males. Those with acute pericarditis were hospitalized in 50% of cases, while patients with myopericarditis were admitted in 97% of cases [8]. In the United States, the average hospitalization rate after ED admission was 72% in the years between 2006 and 2013. Older patients, women, and those with Medicare insurance were more frequently admitted after ED evaluation [4]. According to the 2025 European Society of Cardiology (ESC) guidelines [10], patients with acute pericarditis should be hospitalized in case they present one or more clinical features suggesting a high‐risk presentation, including fever, subacute onset, large pericardial effusion, cardiac tamponade, lack of response to non‐steroidal anti‐inflammatory drugs (NSAIDs) after 7 days, evidence of myopericarditis, immunosuppression, trauma, or ongoing anticoagulation. In the abovementioned studies, however, no or limited information is provided with regard to treatment patterns of patients managed in the ED [4, 5, 6, 7, 8].

Here, we aimed at describing general characteristics, management (including treatment dosing and patterns), and prognosis of patients with acute pericarditis admitted to the ED of a large teaching hospital in Northern Italy.

## 2 Materials and Methods

### 2.1 Study Design

This retrospective, single‐centre study was conducted at the ED of Ospedale di Circolo and Fondazione Macchi (ASST Sette Laghi) in Varese (Italy) from January 1st 2019 to December 31st 2023 among patients with suspected acute pericarditis.

The study (Valutazione della gestione DIagnostica e TErapeutica e della prognosi dei pazienti con Pericardite Acuta afferenti al Pronto soccorso dell'Ospedale di Circolo di Varese, ASST Sette Laghi [Observational Study of Acute Pericarditis in the Varese Emergency Department—OSAP‐VED study]) was conducted in accordance with the 1975 Declaration of Helsinki, as revised in 2024, and approved by the local Institutional Review Board (Comitato Etico Territoriale Lombardia 5, Rozzano, Italy; protocol number 153/24). Due to the retrospective nature of the study, a waiver for the informed consent was provided.

### 2.2 Inclusion and Exclusion Criteria

During the study period, adult patients who were consecutively discharged from the ED with an International Classification of Diseases, 9th revision‐Clinical Modification (ICD‐9‐CM) and/or a descriptive diagnosis on the ED discharge report related to pericarditis or myopericarditis were included (Supplementary Table 1). A thorough chart review was performed by two trained investigators (LC, CL) to double‐check whether the ED discharge diagnosis fulfilled the 2015 ESC criteria for acute pericarditis or myopericarditis [1].

Exclusion criteria were: patients < 18 years of age, diagnosis of acute pericarditis not concordant with the criteria outlined in the 2015 ESC guidelines, and unavailability of electronic medical records (EMRs) through the hospital admission (Supplementary Figure 1).

### 2.3 Clinical Data Collection

Clinical information (including demographic and anthropometric data and vital parameters) and laboratory and imaging findings were collected after reviewing the EMRs through Portale di reparto (Hospital IT System, CBIM) and wHospital InPatient system (wHealth‐Lutech Group, Cinisello Balsamo, Milan, Italy).

Information about the occurrence of complications across the 12‐month follow‐up period was retrieved through Portale di reparto, Hospital IT System (CBIM). Survival status and date of death were gathered from the electronic registry of the Lombardy region (Sistema Informativo Socio‐Sanitario [SISS]).

### 2.4 Endpoints

The primary endpoint was the occurrence of the composite outcome through a 12‐month follow‐up period, including any of the following events (whichever came first): treatment failure, recurrent pericarditis, cardiac tamponade, constrictive pericarditis, or death.

Secondary endpoints included: (i) evaluation of risk factors for the primary endpoint; (ii) evaluation of the therapeutic management in terms of drug prescription and drug doses at the time of ED admission and discharge either from the ED or any hospital divisions; and (iii) evaluation of the prognostic role of troponin T with regard to the primary endpoint.

### 2.5 Definitions

Recurrent pericarditis is defined as the appearance of symptoms and signs of acute pericarditis after a symptom‐free period of 4 to 6 weeks, whereas incessant pericarditis refers to symptoms lasting more than 4–6 weeks but less than 3 months [1]. Constrictive pericarditis is defined by the development of hemodynamic diastolic impairment due to chronic inflammation or thickening of the pericardium diagnosed at echocardiography, cardiac magnetic resonance, or surgical biopsy [1]. Cardiac tamponade is identified by the presence of pericardial effusion leading to hemodynamic compromise, diagnosed with echocardiographic criteria [1]. Treatment failure is defined by the recurrence of symptoms within the first 4 weeks following an initial period of clinical improvement in patients who received any anti‐inflammatory treatments.

Myopericarditis refers to pericarditis with known or clinically suspected concomitant myocardial involvement along with the elevation of markers of myocardial injury (i.e., troponin) [1].

Pericardial effusion is classified as mild (< 10 mm), moderate (10–20 mm), or large (> 20 mm) according to the definition of 2015 ESC guidelines [1].

Percutaneous cardiac procedures (PCPs) include the following: atrial fibrillation (AF) ablation, percutaneous coronary intervention (PCI), and pacemaker (PM) implantation. Cardiac surgery considers coronary artery bypass graft surgery (CABG) and surgical aortic or mitral valve replacement.

The estimated glomerular filtration rate (eGFR) is calculated using the 2021 Chronic Kidney Disease Epidemiology Collaboration (CKD‐EPI) equation [11].

At the time of ED triage, five colour‐coded categories were assigned to patients: white (non‐critical patient; stable condition of minimal clinical relevance), green (minor urgency; non‐evolving, stable condition), yellow (deferrable urgency; non‐evolving, stable condition, with suffering patient), orange (urgency; need for rapid clinical evaluation due to potentially evolving condition with impairment of vital functions), and red (emergency; need for immediate clinical evaluation because of severe impairment of vital functions).

### 2.6 Statistical Analysis

Continuous variables that were normally distributed are expressed as mean with standard deviation or as median with interquartile range if skewed. Categorical variables are reported as counts and percentages (%). Comparisons of continuous variables are performed through Student's *t*‐test or Mann Whitney *U* test, as appropriate, while for categorical variables Fisher's exact or chi‐squared tests are used, as appropriate. Clinically relevant variables and variables significant for *p* ≤ 0.1 at the univariable analysis are entered into a multivariable Cox regression model using a backward stepwise regression to assess the association with the primary composite outcome [12, 13], which is expressed as hazard ratio (HR) with 95% confidence interval (95% CI). This model includes: age, sex, type 2 diabetes, chronic obstructive pulmonary disease (COPD), baseline diastolic blood pressure, haemoglobin, creatinine, C‐reactive protein (CRP), and moderate pericardial effusion, recent PCPs, severe acute respiratory syndrome coronavirus‐2 (SARS‐CoV‐2) vaccination in the prior month and ≥ 2 SARS‐CoV‐2 vaccinations at any time. Missing data are handled through multiple imputations via chained equations in multivariable‐adjusted models to mitigate a missing data bias and ensure robust and reliable estimates (*n* = 20 datasets), and results are pooled across imputations using Rubin's rules [14]. Internal validation is conducted by bootstrapping based on all variables included in the multivariable‐adjusted model, including the outcome variable, to assess model stability and its predictive performance [15]. For all statistical analyses, a two‐tailed *p* < 0.05 is considered statistically significant. Analyses are performed using IBM SPSS Statistics for Mac, version 28.0 (IBM CO., Armonk, NY, USA) and GraphPad Prism, version 8.2 for Windows (GraphPad Software, La Jolla, CA; www.graphpad.com).

## 3 Results

### 3.1 Baseline Characteristics of the Overall Cohort

Out of 354 patients referred to the ED for suspected acute pericarditis, 185 were excluded as they failed to fulfil the 2015 ESC diagnostic criteria [1], and 169 were finally included in the present study. Patients who were excluded differed for age and sex compared with those included.

The median age of the cohort was 54 [40–68] years, with a prevalence of males (*n* = 110, 65.1%) (Table 1). Main comorbidities were hypertension (*n* = 66, 39.1%), coronary artery disease (CAD; *n* = 28, 16.6%), and type 2 diabetes (*n* = 20, 11.8%). Eleven patients (6.6%) suffered from autoimmune diseases, especially systemic lupus erythematosus and rheumatoid arthritis (Table 1).

**TABLE 1:** Baseline characteristics of the overall cohort.

	Number of observations	Overall cohort (*N* = 169)
Demographics		
Age, years	169	54 [40–68]
Sex		
Male, *n* (%)	169	110 (65.1)
Female, *n* (%)		59 (34.9)
Vitals		
SBP, mmHg	156	130 [115–140]
DBP, mmHg	156	80 [70–85]
HR bpm	134	88 [76–100]
SpO_2_, %	144	98 [96–98]
Body temperature, °C	147	36.5 [36.0–37.1]
NRS scale	45	5 [3–8]
BMI, kg/m^2^	87	25.3 [22.4–29.0]
Medical history		
Hypertension, *n* (%)	169	66 (39.1)
Type 2 diabetes, *n* (%)	169	20 (11.8)
CAD, *n* (%)	169	28 (16.6)
Atrial fibrillation, *n* (%)	168	16 (9.5)
Chronic heart failure, *n* (%)	167	9 (5.4)
CKD, *n* (%)	169	14 (8.3)
Obesity, *n* (%)	147	22 (15.0)
COPD, *n* (%)	169	8 (4.7)
Autoimmune disease, *n* (%)	169	11 (6.5)
Active cancer, *n* (%)	169	2 (1.2)
Previous cancer, *n* (%)	168	14 (8.3)
Active smoking, *n* (%)	141	42 (29.8)
Prior radiotherapy, *n* (%)	166	5 (3.0)
Recent chest trauma, *n* (%)		0 (0)
Recent PCPs, *n* (%)		11 (6.5)
PCI and stenting, *n* (%)	169	8 (4.7)
PM implantation/cardiac ablation, *n* (%)		3 (1.8)
Recent cardiac surgery, *n* (%)	169	3 (1.8)
Laboratory findings		
WBC, ×10^9^/L	168	10.5 [8.4–12.8]
Haemoglobin, g/dL	168	13.8 [12.6–14.9]
Platelets, ×10^9^/L	168	236 [200–300]
Creatinine, mg/dL	167	0.9 [0.8–1.1]
eGFR, mL/min/1.73 m^2^	167	91 [72–105]
CRP, mg/L	159	33.3 [6.5–84.0]
Troponin T, ng/L	162	13.0 [5.0–35.5]
NT‐proBNP, pg/ml	46	275 [141–1158]
LDH, U/L	144	184.5 [159.0–220.0]

Abbreviations: ALT, alanine transaminase; AST, aspartate aminotransferase; BMI, body mass index; CAD, coronary artery disease; CKD, chronic kidney disease; COPD, chronic obstructive pulmonary disease; CRP, C‐reactive protein; DBP, diastolic blood pressure; eGFR, estimated glomerular filtration rate; HR, heart rate; LDH, lactate dehydrogenase; NRS, numeric rating scale; NT‐proBNP, N‐terminal pro‐B‐type natriuretic peptide; PCI, percutaneous coronary intervention; SBP, systolic blood pressure; SpO2, saturation of peripheral oxygen; WBC, white blood cell.

At ED admission, the median value of white blood cells (WBC) was 10.5 [8.4–12.8] x10^9^/L, whereas the median value of CRP was 33.3 [6.5–84.0] mg/L. In the whole cohort, 44 patients (27.7%) presented with CRP values ≤ 10 mg/L. Median troponin T was 13 [5.5–35.5] ng/L (normal values: < 14 ng/L), with 46.3% of patients (*n* = 75) presenting with an abnormal value (Table 1).

### 3.2 Clinical Presentation at the ED Admission and Admission/Discharge Patterns

Within the whole cohort, 136 patients (80.5%) had a first episode of acute pericarditis, 32 (19.2%) presented with recurrent pericarditis, and 1 (0.6%) with incessant pericarditis. Myopericarditis occurred in 74 patients (44.6%).

**FIGURE 1:**
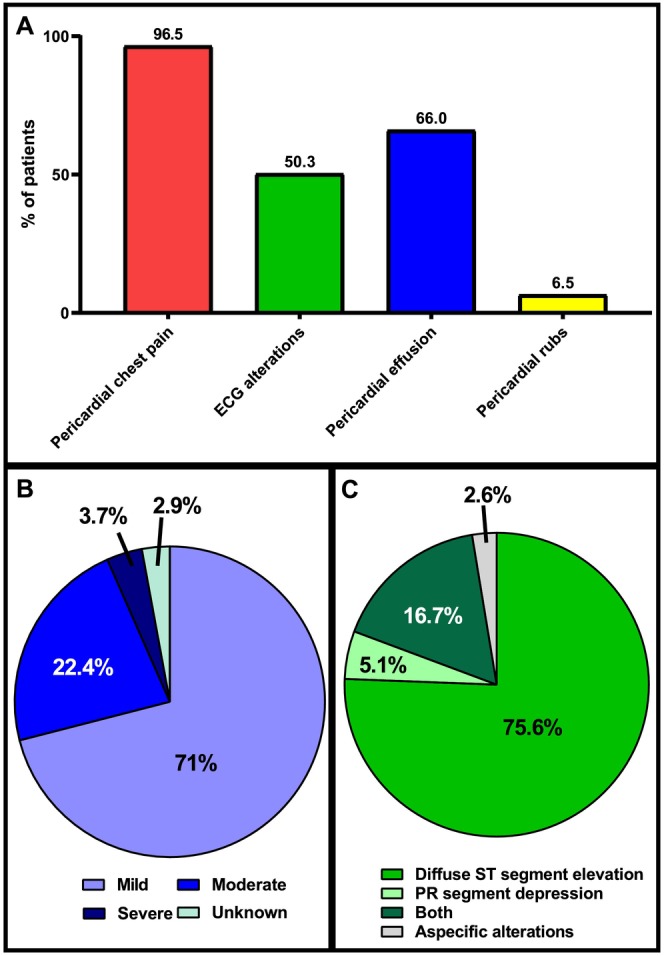
Clinical presentation at the emergency department. Panel A: prevalence of signs and symptoms of acute pericarditis. Panel B: size of the pericardial effusion. Panel C: specific ECG modifications.

The most frequent symptoms at ED admission were pericardial chest pain (*n* = 163, 96.4%) and pericardial effusion (*n* = 107, 66.0%) (Figure 1A), the latter being mild in most cases (Figure 1B). In addition, more than half of patients (*n* = 78) presented with electrocardiogram (ECG) modifications, mainly diffuse ST segment elevation (Figure 1C). In 9 patients (5.5%), a first episode of atrial fibrillation (AF) was documented.

Similar proportions of patients presented to the ED across the four seasons of the year (Supplementary Figure 2A), mainly from home (Supplementary Figure 2B), self‐presented (Supplementary Figure 2C), and were assigned a green or yellow code at ED triage (Supplementary Figure 2D).

Out of 169 patients, 60 (35.5%) were discharged home, with a median ER length of stay of 1 [0–1] day. One hundred and nine patients (64.5%) were hospitalized in the Cardiology (*n* = 53, 48.6%), Internal Medicine (*n* = 30, 27.5%), and Emergency Medicine (*n* = 12, 11.0%) divisions with a median length of stay of 6 [4–10] days.

### 3.3 Predisposing Factors

A viral infection was reported by 65 patients (38.5%) in the month prior to the ED admission: 49 (29.2%) experienced a flu‐like syndrome and 18 (10.7%) acute gastroenteritis. Patients who already got ≥ 2 vaccinations for SARS‐CoV‐2 were 80 (47.3%), and, among them, 12 (7.1%) received their last dose in the month prior to ED admission (Supplementary Table 2).

With regard to PCPs performed within 3 months from the index event, 11 patients (6.5%) received PCI and stent implantation for acute coronary syndrome, while a small portion of patients underwent PM implantation or AF ablation and cardiac surgery (Table 1).

### 3.4 Treatment Patterns

**FIGURE 2:**
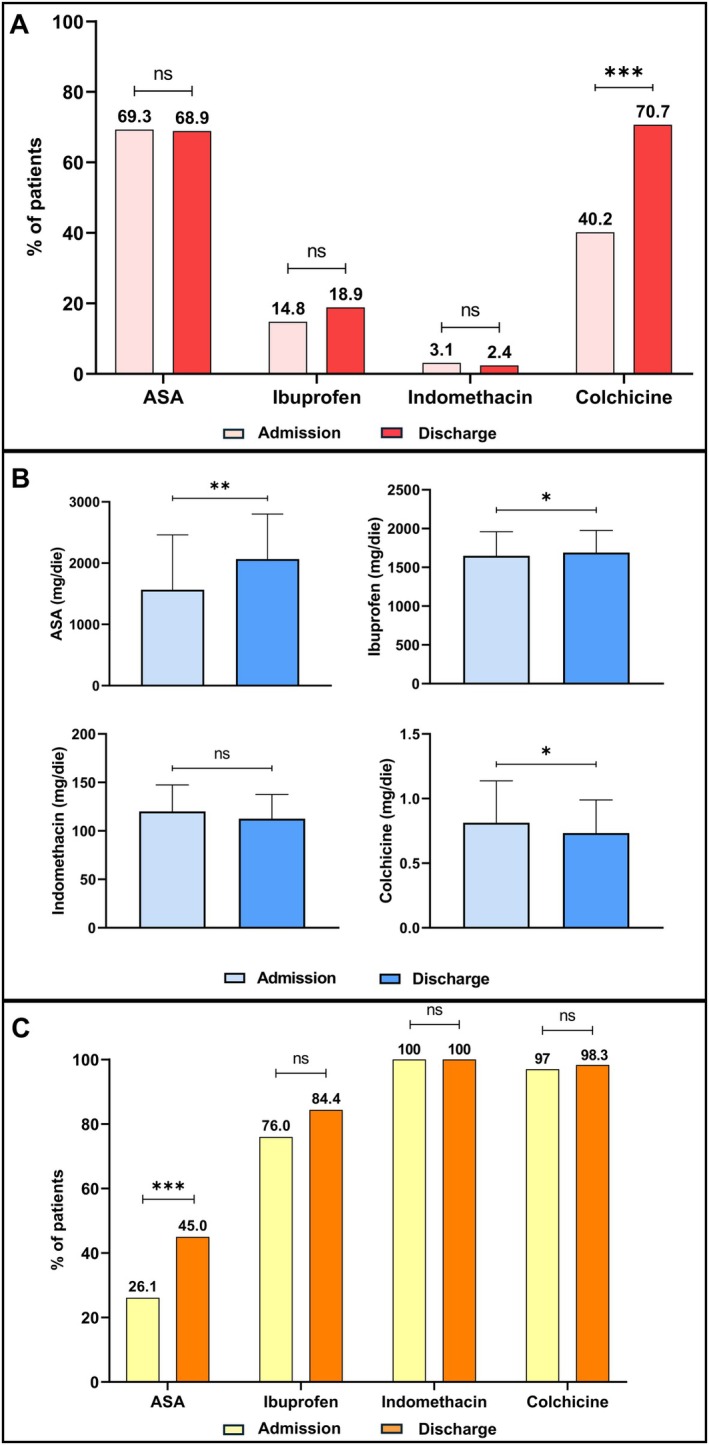
Pharmacological treatment at admission and discharge. Panel A: proportion of prescribed guideline‐directed drugs at admission and discharge. Panel B: mean daily doses of guideline‐directed drugs at admission and discharge. Panel C: proportion of patients on suggested doses of guideline‐directed drugs at admission and discharge. *p = 0.01; **p < 0.01; ***p < 0.001; ns = non‐statistically significant.

At the time of diagnosis in the ED, aspirin was prescribed in 113 patients (69.3%) and NSAIDs in 32 patients (19.5%), including ibuprofen (*n* = 25, 14.8%) and indomethacin (*n* = 5, 3.0%) (Figure 2A). Colchicine was prescribed in 66 patients (40.2%) (Figure 2A). Nine patients (5.5%) were given prednisone.

At discharge (either from the ED or from a hospital division), the proportion of patients on aspirin remained unchanged (*n* = 115, 68.9%), while a larger proportion of patients were prescribed ibuprofen (18.9%, *n* = 32). A slight reduction in the prescription of indomethacin was observed (2.4%, *n* = 4) (Figure 2A). On the contrary, patients discharged on colchicine significantly increased (70.7%, *n* = 118; *p* < 0.001) (Figure 2A). Also, the prescription of prednisone increased at the time of hospital discharge (12%, *n* = 20) (Supplementary Figure 3A). A general increase in the daily dose of the drugs from admission to discharge was observed for aspirin (from 1567 ± 894 mg to 2066 ± 736 mg, *p* = 0.001) and ibuprofen (from 1648 ± 312 mg to 1691 ± 284 mg, *p* = 0.023), while a dose reduction occurred for colchicine (from 0.81 ± 0.32 mg to 0.73 ± 0.26 mg, *p* = 0.012) and indomethacin (from 120 ± 27 mg to 113 ± 25 mg, *p* = ns) (Figure 2B). A dose reduction was observed for prednisone (from 12 ± 16 mg to 26 ± 20 mg, *p* < 0.001) (Supplementary Figure 3B).

At ED admission, guideline‐directed drug doses were prescribed to 26.1% (*n* = 29) of patients on aspirin, 76% (*n* = 19) of those on ibuprofen, to all patients on indomethacin (*n* = 5, 100%), and to 97% of those on colchicine (*n* = 65, 97%) (Figure 2C and Supplementary Table 3). At discharge, a statistically significant increase in the proportion of patients on guideline‐directed doses was observed for aspirin (45%, *n* = 50; *p* < 0.001), but not for ibuprofen (84%, *n* = 27; *p* = ns). Similarly, no significant differences were found for those on indomethacin (*n* = 4, 100%; *p* = ns) and colchicine (*n* = 116, 98.3%; *p* = ns), who were already for the large majority on the correct dose of each drug (Figure 2C). Since weight was not available for all patients, this assessment cannot be made for prednisone (guidelines suggest to prescribe glucocorticoids at a dosage of 0.2 to 0.5 mg/kg daily [1, 10]).

In the whole cohort, few patients presented allergies to guideline‐directed treatments: 6 patients (3.6%) were allergic to aspirin, and 3 (1.8%) to ibuprofen. No patient reported any allergy to indomethacin and colchicine.

### 3.5 Composite Outcome at 12 Months

The composite outcome occurred in 34 patients (20.1%), with recurrences and treatment failure representing the most common events. Throughout the follow‐up, 18 patients (11.2%) experienced one recurrence, 7 patients (4.1%) 2 recurrences, and 2 patients (1.2%) 3 recurrences. Neither cardiac tamponade nor constrictive pericarditis was recorded.

Compared to uncomplicated patients, those who experienced the composite outcome at 12 months were older (60 vs. 52 years), more frequently females (50% vs. 31%), and suffered from type 2 diabetes (26.5% vs. 8.1%) and COPD (14.7% vs. 2.2%). They also present with lower haemoglobin (12.9 vs. 13.9 g/dL) and higher CRP (61.2 vs. 32.2 mg/L) levels (Table 2). No differences in terms of clinical presentation were recorded (Supplementary Table 4). Among predisposing factors, recent PCPs were more frequently observed in complicated patients (14.7% vs. 4.4%, *p* = 0.030) (Table 2) and the same occurred with SARS‐CoV‐2 vaccination in the month prior to acute pericarditis (11.8% vs. 2.2%, *p* = 0.012) (Supplementary Table 5). A trend toward an increase in the occurrence of the primary endpoint was observed for ibuprofen and colchicine (Supplementary Figure 4) when drug doses were not in line with guideline recommendations at discharge (Supplementary Table 3).

**TABLE 2:** Baseline characteristics of the overall cohort based on the occurrence of the 12‐month composite outcome.

	Uncomplicated cases (*N* = 135)	Patients with composite outcome (*N* = 34)	*p*
Demographics			
Age, years	52 [38–67]	60 [43–74]	0.071
Sex			
Male, *n* (%)	93 (68.9)	17 (50.0)	0.046
Female, *n* (%)	42 (31.1)	17 (50.0)	
Vitals			
SBP, mmHg	130 [117–142]	126 [110–140]	0.157
DBP, mmHg	80 [70–85]	70 [60–80]	0.015
HR, bpm	89 [77–101]	81 [74–96]	0.316
SpO_2_, %	98 [96–99]	97 [95–98]	
Body temperature, °C	36.5 [36.0–37.0]	36.3 [36.0–37.3]	0.842
NRS scale	6 [3–7]	5 [2–8]	0.922
BMI, kg/m^2^	24.8 [21.9–28.9]	26.4 [24.6–29.1]	0.185
Medical history			
Hypertension, *n* (%)	52 (38.5)	14 (41.2)	0.845
Type 2 diabetes, *n* (%)	11 (8.1)	9 (26.5)	0.006
CAD, *n* (%)	21 (15.6)	7 (20.6)	0.451
Atrial fibrillation, *n* (%)	12 (9.0)	4 (11.8)	0.743
Chronic heart failure, *n* (%)	6 (4.5)	3 (9.1)	0.382
CKD, *n* (%)	12 (8.9)	2 (5.9)	0.738
Obesity, *n* (%)	17 (14.5)	5 (16.7)	0.777
COPD, *n* (%)	3 (2.2)	5 (14.7)	0.009
Autoimmune disease, *n* (%)	8 (5.9)	3 (8.8)	0.464
Active cancer, *n* (%)	2 (1.5)	—	> 0.99
Previous cancer, *n* (%)	11 (8.2)	3 (8.8)	0.808
Active smoking, *n* (%)	33 (30.0)	9 (29.0)	0.807
Prior radiotherapy, *n* (%)	4 (3.0)	1 (3.1)	> 0.99
Recent chest trauma, *n* (%)	—	—	—
Recent PCPs, *n* (%)	6 (4.4)	5 (14.7)	0.030
PCI and stenting, *n* (%)	3 (2.2)	5 (14.7)	0.009
PM implantation/cardiac ablation, *n* (%)	3 (2.2)	—	> 0.99
Recent cardiac surgery, *n* (%)	3 (2.2)	—	> 0.99
Laboratory findings			
WBC, ×10^9^/L	10.4 [8.3–12.8]	11.1 [8.7–13.2]	0.418
Haemoglobin, g/dL	13.9 [12.8–15.1]	12.9 [11.6–14.2]	0.008
Platelets, ×10^9^/L	237 [201–304]	226 [195–275]	0.291
Creatinine, mg/dL	1.0 [0.8–1.1]	0.9 [0.8–1.0]	0.043
eGFR, mL/min/1.73 m^2^	91 [73–105]	86 [68–105]	0.709
CRP, mg/L	32.2 [5.6–74.0]	61.2 [13.3–128.9]	0.033
Troponin T, ng/L	11.5 [4.8–35.5]	16.0 [8.0–42.2]	0.120
NT‐proBNP, pg/mL	269.5 [122.0–1076.8]	391.5 [170.2–2360.5]	0.305
LDH, U/L	185 [155–220]	182 [165–217]	0.887

Abbreviations: ALT, alanine transaminase; AST, aspartate aminotransferase; BMI, body mass index; CAD, coronary artery disease; CKD, chronic kidney disease; COPD, chronic obstructive pulmonary disease; CRP, C‐reactive protein; DBP, diastolic blood pressure; eGFR, estimated glomerular filtration rate; HR, heart rate; LDH, lactate dehydrogenase; NRS, numeric rating scale; NT‐proBNP, N‐terminal pro‐B‐type natriuretic peptide; PCI, percutaneous coronary intervention; SBP, systolic blood pressure; SpO_2_, saturation of peripheral oxygen; WBC, white blood cell.

**FIGURE 3:**
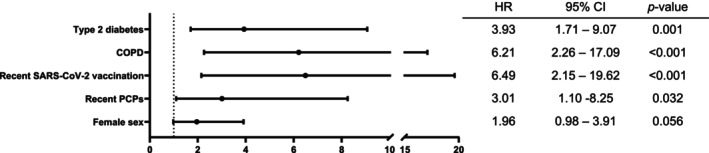
Risk factors for the primary endpoint. The forest plot shows conditions that independently increased the occurrence of the primary composite outcome (treatment failure, recurrent pericarditis, cardiac tamponade, constrictive pericarditis, or death across the 12‐month follow‐up period). CI, confidence interval; COPD, chronic obstructive pulmonary disease; HR, hazard ratio; PCP, percutaneous cardiac procedure; SARS‐CoV‐2, severe acute respiratory syndrome coronavirus‐2.

Based on a Cox proportional hazard model with backward stepwise elimination, the presence of type 2 diabetes, COPD, recent PCPs, and SARS‐CoV‐2 vaccination in the prior month were independently associated with the occurrence of the composite outcome (Figure 3). In addition, female sex showed a trend toward a doubled risk of complications but did not reach statistical significance (Figure 3).

### 3.6 Exploratory Analyses for the Composite Outcome at 12 Months

In spite of the potential heterogeneity in the aetiologies of acute pericarditis, the limited sample size and the relatively small number of patients within specific etiologic subgroups did not allow for adequately powered, robust subgroup analyses. We have then conducted a sensitivity analysis excluding major secondary causes (recent PCPs, recent cardiac surgery, prior radiotherapy; *n* = 18), and we have found that COPD and recent SARS‐CoV‐2 vaccination showed a trend for an increased risk of the composite outcome, while haemoglobin looked protective (Supplementary Table 6). It is important, however, to acknowledge the broad confidence interval for COPD and recent SARS‐CoV‐2 vaccination when interpreting this sub‐analysis, which should be considered as exploratory.

Since the composite outcome was mostly driven by recurrences, we have performed a secondary analysis focused on recurrences only. Given the limited number of recurrences (*n* = 18), multivariable modelling was restricted to a small number of clinically selected covariates to avoid overfitting, i.e., variables that were statistically significant in the univariable analysis and clinically relevant variables and/or variables described in the literature to be associated with an increased rate of recurrences (female sex, recent percutaneous procedures, baseline use of prednisone and colchicine, prior episode of acute pericarditis). Two different models resulted, suggesting that ECG changes, increasing levels of CRP, female sex, and recent PCPs may increase the risk for recurrences (Supplementary Table 7). Due to the lower number of recurrences, the results of these analyses should be considered as exploratory.

### 3.7 Troponin T at ED Admission

Median troponin T at the time of ED admission was 13.0 [5.0–35.5] ng/L (Table 1), which is considered normal according to reference values of the Central Laboratory of our Institution (< 14 ng/L). Troponin T was not measured in 7 patients (4.1%).

Patients with elevated troponin T (≥ 14 ng/L), i.e., with myopericarditis, were older (62 vs. 51 years) and more frequently suffered from hypertension (53.3% vs. 27.6%), type 2 diabetes (21.3% vs. 4.6%), chronic heart failure (9% vs. 0%), chronic kidney disease (16.0% vs. 2.3%) (Supplementary Table 8). In addition, they presented with lower haemoglobin levels (13.4 vs. 14.1 g/dL), platelet count (220 vs. 261 × 10^9^/L), estimated glomerular filtration rate (86 vs. 94.5 mL/min/1.73 m^2^) and higher CRP (58.4 vs. 22.4 mg/L), NT‐proBNP (635 vs. 182 pg/mL), and LDH (199 vs. 177.5 U/L) values (Supplementary Table 8). No differences between the two groups were observed with regard to clinical presentation (Supplementary Table 9) and predisposing factors (Supplementary Table 10). A higher, although not statistically significant, proportion of patients with myopericarditis experience the 12‐month composite outcome compared to those with pericarditis only (24% [*n* = 18] vs. 16.1% [*n* = 14], *p* = 0.238), while no clear differences were observed with regard to recurrences (8 [10.7%] vs. 9 [10.3%], *p* = 0.947).

## 4 Discussion

Given the limited evidence on the management of pericarditis in the ED, this study aimed to expand current knowledge in this context. In our study, main findings can be summarized as follows: (i) a significant percentage of patients with acute pericarditis experienced long‐term complications, especially recurrences; (ii) type 2 diabetes, COPD, recent PCPs and SARS‐CoV‐2 vaccination in the month prior to the ED admission were identified as independent risk factors for adverse outcomes; (iii) patients with abnormal troponin T experienced a higher, not statistically significant proportion of complications; (iv) treatment patterns were not always adherent to guideline recommendations.

The prevalence of the male sex in our cohort as well as of common comorbidities (diabetes, hypertension, and CAD) align with data from other European cohorts [16], but differ from what reported in a United States population [17], likely reflecting epidemiological differences. Autoimmune diseases were observed in less than 10% of patients, as previously reported [17, 18]. Elevation of CRP (> 10 mg/L) was recorded in a substantial percentage of our patients (72.3%), and is in line with guidelines [1, 19] and prior studies [17, 20, 21]. Mascolo et al. described a subset of patients with recurrent pericarditis with normal CRP concentrations in the proportion of 25% [22], which is similar to what observed in our cohort. This finding highlights the presence of a non‐inflammatory phenotype, with specific clinical features and direct consequences on the therapeutic management, as highlighted elsewhere [23] that is worth being further investigated. Despite not being a major criterion for the diagnosis according to 2015 ESC guidelines [1], recent ESC guidelines [10] and an expert consensus by the American College of Cardiology [19] both support the role of CRP along with pericardial chest pain and cardiac imaging to achieve a definite diagnosis of acute pericarditis, especially in patients with an inflammatory phenotype [21, 24].

In terms of clinical presentation, pericardial chest pain was the most frequent symptom, in accordance with the published literature [6, 7, 8]. Electrocardiographic changes were recorded in similar proportions to others [6, 7, 8], while the prevalence of pericardial effusion was higher in our cohort probably depending either on the prompt availability of echocardiographic assessment in the ED or on the early presentation of patients with a high inflammatory burden. In addition, we found that 5.5% of patients experienced a first episode of AF at the time of ED admission, while previous studies reported higher proportions [16, 25, 26]. This finding should not be surprising as acute pericardial inflammation represents an arrhythmogenic trigger for supraventricular arrhythmias [27, 28], as previously reported [29, 30]. Importantly, a new diagnosis of atrial fibrillation poses a dilemma with regard to anticoagulation mainly due to an increased risk of bleeding when associated with aspirin and/or NSAIDs. In addition, oral anticoagulation was reported as a minor predictor of poor prognosis in acute pericarditis [10].

Among predisposing factors, more than two‐thirds of patients reported symptoms consistent with a viral infection in the previous month, supporting the hypothesis that viruses may act as non‐specific triggers of pericardial inflammation [2, 3, 18, 31]. With regard to SARS‐CoV‐2 vaccination, only 7.1% of patients received a dose of vaccine in the 30 days before the occurrence of acute pericarditis. Post‐SARS‐CoV‐2 vaccine pericarditis was described as generally self‐limited, with some rare, complex forms [32, 33]. However, it is important to underline that in our cohort SARS‐CoV‐2 vaccination was associated with complications in a very small portion of patients. Due to the small numbers and the wide confidence intervals, causality cannot be inferred, and the results of this analysis should be considered as exploratory. In addition, evidence from a large retrospective population‐based cohort study in the Lombardy region (Italy) confirmed the clinical benefits of SARS‐CoV‐2 vaccination over the rare occurrence of cardiovascular complications and their good prognosis [32]. A small group of patients experienced acute pericarditis following cardiac procedures that represent an important risk factor, as already highlighted by other authors [17, 18, 34, 35]. Since cardiac procedures are being increasingly performed in past years, post‐pericardiotomy syndrome represents a frequent cause of acute pericarditis in developed countries [36]. Pathophysiological mechanisms rely on the activation of the immune system by the pericardial injury itself that drives local inflammation through inflammasome activation. Finally, despite not being statistically significant, female sex was likely to represent a factor associated with poor prognosis, although this finding is controversial [37, 38, 39].

Therapeutic management of patients admitted to the ED with acute pericarditis generally followed contemporary guidelines, although some discrepancies emerged with respect to suggested doses of the drugs. Aspirin was more frequently prescribed than NSAIDs (ibuprofen, indomethacin). Actually, neither aspirin nor NSAIDs have been proved to be superior to one another while associated with colchicine [40, 41]. Since aspirin is preferred in patients with CAD and is considered safer than NSAIDs for patients with chronic kidney disease, heart failure and hypertension [42, 43, 44], its preferential use in our cohort may be due to the high prevalence of these comorbidities. Colchicine was prescribed in only 41% of patients at admission, however this proportion significantly increased to 71% at discharge, suggesting that colchicine was introduced later in the treatment plan, for example, at the time of ED discharge. Indeed, strong evidence supports its early prescription to reduce the risk of recurrences [41]. In terms of drug doses, aspirin was prescribed at lower doses than suggested by guidelines both at admission and discharge in more than 50% of patients, despite guidelines stress the importance of using full‐dose anti‐inflammatory therapies [18, 45]. On the contrary, doses of ibuprofen, indomethacin and colchicine complied with those suggested by the guidelines. A reason for such disparity could be found in the general fear of physicians and/or patients about side effects of high‐dose aspirin, although NSAIDs are burdened by similar side effects when given at full doses without the prescription of proton pump inhibitors. Another, not negligible aspect deals with patient's preferences about lower doses of NSAIDs or aspirin, that may favour adherence to therapy, although this may increase the time needed to control acute pain driven by the underlying inflammation of the pericardium. Importantly, our findings appear poorly comparable with the available literature, since all studies considered did not report the daily dose of NSAIDs and aspirin prescribed to patients at the time of the diagnosis.

The primary endpoint occurred in 20.1% of patients, and recurrences were the most frequent events, similarly to prior studies [17, 18, 26, 46]. This occurred more frequently in older patients, of female sex, and in those with comorbidities—type 2 diabetes and COPD that is partially concordant with previous findings [8, 17, 26]. The association between female sex and adverse outcomes may reflect the higher prevalence of autoimmune etiologies in women, potentially driven by sex‐based differences in immune regulation and antibody production, which could favour a hyperimmune response [38]. Compared to uncomplicated cases, patients who experienced complications exhibited higher CRP and lower hemoglobin levels, suggesting a larger inflammatory burden underlying the auto‐inflammatory phenotype of such patients [21, 24]. Importantly, increased levels of CRP, female sex, and recent PCPs are likely to increase the risk of recurrences in our cohort [17, 20], confirming the existing literature, although these analyses should be considered as exploratory due to the limited sample size.

In our study, troponin T was elevated in 45.6% of patients, as already reported [17, 25, 47, 48]. Older age, type 2 diabetes, heart failure, and chronic kidney disease were more frequently associated with myocardial involvement, and this is partially concordant with what was found by Vecchié et al. [17] but different from Imazio et al. [25], while other studies did not report specifically differences in comorbidities based on troponin elevation [25, 48]. In contrast with others [17, 48], we found that troponin T elevation was associated with a slightly higher, albeit not significant, proportion of complications, but this was not true for recurrences. Reasons for such differences may depend on the different composition and origin of the cohorts, on specific clinical presentations, and on a larger burden of cardiovascular diseases in our cohort. However, troponin increase in patients with myopericarditis is usually mild and highlights the involvement of the myocardium, especially when coexisting with ECG changes [2, 3].

Our study presents many strengths, such as the high diagnostic accuracy and the long‐term follow‐up (12 months). Patients were included in the study after a thorough review of medical records to confirm that those assigned with ICD‐9‐CM codes related to pericardial diseases really met the diagnostic criteria for acute pericarditis defined by the 2015 ESC guidelines [1]. As a result, only 169 out of 354 patients had a confirmed diagnosis of acute pericarditis. This aspect is of utmost importance since diagnostic misclassification and inaccuracy are frequent when conducting retrospective studies using ICD‐9‐CM codes for pericardial diseases [49]. While reading our paper, limitations should also be acknowledged. Despite using unbiased tools for the inclusion of patients, the retrospective nature of a single‐centre study enrolling a limited number of subjects may retain residual selection bias and ascertainment bias related to missing data, thus limiting the generalization of the results. In addition, by only using ICD‐9‐CM codes, atypical cases or missed diagnoses could have been excluded, as well as complications managed in hospitals outside our healthcare network cannot be tracked since follow‐up phone calls or access to medical records of the patients were not possible. As the population carried a high burden of comorbidities needing hospitalization, this may prevent generalizing our results to younger populations with few or no comorbidities who can be managed in the outpatient setting. The inclusion of patients with pericarditis and myopericarditis might influence comparability with other cohorts. However, apart from an increased proportion of cardiovascular conditions, that was expected, no differences either in terms of clinical presentation nor of predisposing factors were found in our cohort. In addition, the latest ESC guidelines introduced the novel concept of inflammatory myopericardial syndromes to encompass the large spectrum ranging from pericarditis to myocarditis and the coexistence of these two conditions [10]. Regarding this, we should acknowledge the potential impact of etiologic heterogeneity on the observed association with the composite outcome. Given the limited sample size and the relatively small number of patients within specific etiologic subgroups that prevented adequately powered subgroup analyses, we have conducted a sensitivity analysis excluding major secondary causes. Finally, given the study design, it was not possible to collect any detailed information either about complications occurred during the hospital stay nor regarding side effects for NSAIDs and colchicine.

In conclusion, a significant proportion of patients with acute pericarditis experiences long‐term complications, in particular recurrent pericarditis, and those with type 2 diabetes, COPD, recent PCPs, and a recent SARS‐CoV‐2 vaccination are at higher risk for adverse outcomes. With regard to recent SARS‐CoV‐2 vaccination, it should be acknowledged that this event occurred in a very small portion of patients and that findings from a prior report support the clinical benefits of SARS‐CoV‐2 vaccination over the rare occurrence of cardiovascular complications. Our study suggests the importance of a larger adherence to guideline recommendations regarding the doses of anti‐inflammatory drugs and the early prescription of colchicine, particularly related to complications (i.e., recurrences). Underdosing of drugs is likely to play a role in complications; however, this finding should be considered exploratory given the retrospective nature of our study. Future studies should investigate whether an early evaluation of patients in the ED by physicians specialized in managing patients with acute/recurrent pericarditis could reduce the burden of complications by establishing a close follow‐up in the immediate period after discharge. This could translate into an early discharge from the ED and an early prescription of targeted treatments (e.g., IL‐1 blockers) in those patients with clinical features predisposing to a complicated course, such as those with an auto‐inflammatory phenotype.

## Author Contributions

A.B., W.A., L.C. and C.L. conceived the general structure of the study. L.C. and C.L. performed the chart review. L.C., C.L. and A.B. analysed data, wrote the first draft of the manuscript and drafted its revised versions. All authors critically revised the different versions of the manuscript and approved its final version. L.C., C.L. and A.B. are the guarantors of this work and, as such, have full access to all data in the study and take responsibility for the integrity of data and the accuracy of data analysis. L.C. and C.L. equally contributed to the manuscript as first authors.

## Funding

The authors have nothing to report.

## Ethics Statement

The study (Valutazione della gestione DIagnostica e TErapeutica e della prognosi dei pazienti con Pericardite Acuta afferenti al Pronto soccorso dell'Ospedale di Circolo di Varese, ASST Sette Laghi [Observational Study of Acute Pericarditis in the Varese Emergency Department—OSAP‐VED study]) was approved by the local Institutional Review Board (Comitato Etico Territoriale Lombardia 5, Rozzano, Italy; protocol number 153/24). Due to the retrospective nature of the study, a waiver for the informed consent was provided.

## Conflicts of Interest

The authors declare no conflicts of interest.

## Supporting information


**Supplementary Table 1.** Description of ICD‐9‐CM and corresponding ICD‐10‐CM codes.
Supplementary Table 2. Predisposing factors for acute pericarditis in the overall cohort.
Supplementary Table 3. Pharmacological therapy with dosing and duration for acute and recurrent pericarditis as per ESC guidelines.
Supplementary Table 4. Clinical presentation of the overall cohort based on the occurrence of the 12‐month composite outcome.
Supplementary Table 5. Predisposing factors for acute pericarditis based on the occurrence of the 12‐month composite outcome.
Supplementary Table 6. Sensitivity analysis for the composite endpoint excluding major secondary causes.
Supplementary Table 7. Sensitivity analysis for the composite endpoint restricted to recurrences only.
Supplementary Table 8. Baseline characteristics of the overall cohort based on troponin T levels at the time of ED admission.
Supplementary Table 9. Clinical presentation of the overall cohort based on troponin T levels at the time of ED admission.
Supplementary Table 10. Predisposing factors for acute pericarditis based on troponin T levels at the time of ED admission.
Supplementary Figure 1. Participant flow of the OSAP‐VED study.
Supplementary Figure 2. Characteristics of presentation to the emergency department.
Supplementary Figure 3. Prescription of prednisone and doses.
Supplementary Figure 4. Proportion of the primary endpoint according to guideline‐recommended drug doses at discharge.

## Data Availability

The data that support the findings of this study are available from the corresponding author upon reasonable request.
