# Supplementary material for: Management and Prognosis of Patients With Acute Pericarditis in the Emergency Department: A Retrospective, Single‐Centre Study
Source: Eur J Clin Invest. 2026 Apr 24;56:e70206. doi: 10.1111/eci.70206 (PMC13109608; doi:10.1111/eci.70206)
Supplement: Supplementary file 1 — Supplementary Table 1. Description of ICD‐9‐CM and corresponding ICD‐10‐CM codes. Supplementary Table 2. Predisposing factors for acute pericarditis in the overall cohort. Supplementary Table 3. Pharmacological therapy with dosing and duration for acute and recurrent pericarditis as per ESC guidelines. Supplementary Table 4. Clinical presentation of the overall cohort based on the occurrence of the 12‐month composite outcome. Supplementary Table 5. Predisposing factors for acute pericarditis based on the occurrence of the 12‐month composite outcome. Supplementary Table 6. Sensitivity analysis for the composite endpoint excluding major secondary causes. Supplementary Table 7. Sensitivity analysis for the composite endpoint restricted to recurrences only. Supplementary Table 8. Baseline characteristics of the overall cohort based on troponin T levels at the time of ED admission. Supplementary Table 9. Clinical presentation of the overall cohort based on troponin T levels at the time of ED admission. Supplementary Table 10. Predisposing factors for acute pericarditis based on troponin T levels at the time of ED admission. Supplementary Figure 1. Participant flow of the OSAP‐VED study. Supplementary Figure 2. Characteristics of presentation to the emergency department. Supplementary Figure 3. Prescription of prednisone and doses. Supplementary Figure 4. Proportion of the primary endpoint according to guideline‐recommended drug doses at discharge. [file ECI-56-e70206-s001.docx]

**Supplementary Table 1. Description of ICD-9-CM and corresponding ICD-10-CM codes**

| **ICD-9-CM** | **ICD-10-CM** | **Conditions** |
| --- | --- | --- |
| 074.21 | B33.23 | Coxsackie pericarditis |
| 391.0 | I01.0 | Acute rheumatic pericarditis |
| 391.2 | I01.2 | Acute rheumatic myocarditis |
| 393 | I09.2 | Chronic rheumatic pericarditis |
| 411.0 | I24.1 | Postmyocardial infarction syndrome/ Dressler's syndrome |
| 420.0 | I32 | Acute pericarditis in diseases classified elsewhere |
| 420.9 | –––––– | Other and unspecified acute pericarditis |
| 420.90 | I30.9 | Acute pericarditis, unspecified |
| 420.91 | I30.0 | Acute idiopathic pericarditis/ Acute nonspecific idiopathic pericarditis |
| 420.99 | I30.8 | Other acute pericarditis/ Other forms of acute pericarditis |
| 422.0 | I41 | Acute myocarditis in diseases classified elsewhere |
| 422.90 | I40.9 | Acute myocarditis, unspecified |
| 422.92 | I40.0 | Infective myocarditis |
| 422.93 | I40.8 | Toxic myocarditis |
| 422.99 |  | Other acute myocarditis |
| 423.0 | –––––– | Acute rheumatic pericarditis |
| 423.1 | I31.2 | Hemopericardium/ Hemopericardium, not elsewhere classified |
| 423.2 | I31.1 | Constrictive pericarditis/ Chronic constrictive pericarditis |
| 423.8 | I31.8 | Other specified diseases of pericardium |
| 423.9 | I31.9 | Unspecified disease of pericardium |

**Supplementary Table 2. Predisposing factors for acute pericarditis in the overall cohort.**

|  | **Overall cohort (N=169)** |
| --- | --- |
| Any viral infection in the past month, n (%) | 65 (38.5) |
| Flu-like syndrome, n (%) | 49 (29.2) |
| Gastroenteritis, n (%) | 18 (10.7) |
| SARS-CoV-2 infection |  |
| Previous (any time), n (%) | 22 (13.0) |
| Current, n (%) | 1 (0.6) |
| 1 week before, n (%) | 1 (0.6) |
| 1 month before, n (%) | 2 (1.2) |
| 2 to 6 months before, n (%) | 10 (5.9) |
| 7 to 12 months before, n (%) | 3 (1.8) |
| >12 months before, n (%) | 1 (0.6) |
| SARS-CoV-2 vaccination |  |
| Type of vaccine |  |
| Comirnaty (Pfizer- BioNTech), n (%) | 43 (25.4) |
| Spikevax (Moderna), n (%) | 34 (20.1) |
| Vaxzevria (AstraZeneca), n (%) | 3 (1.8) |
| ≥2 doses of any vaccine, n (%) | 80 (47.3) |
| Time between vaccination and ED admission |  |
| 1 week, n (%) | 5 (3.0) |
| 1 month, n (%) | 12 (7.1) |
| 2 to 6 months, n (%) | 25 (14.8) |
| 7 to 12 months, n (%) | 27 (16.0) |
| >12 months, n (%) | 15 (8.9) |
| Other vaccines in the previous month, n (%) | 0 (0) |

Legend. ED: emergency department. PCI: percutaneous coronary intervention. PCP: percutaneous cardiac procedure. PM: pacemaker. SARS-CoV-2: severe acute respiratory syndrome coronavirus-2.

**Supplementary Table 3. Pharmacological therapy with dosing and duration for acute and recurrent pericarditis as per ESC guidelines.**

| **Therapy** | **Dosing** | **Duration** | **Tapering** |
| --- | --- | --- | --- |
| Aspirin | 750-1,000 mg 3 times daily | 1 to 2 weeks | Decrease by 250 mg every 1-2 weeks |
| Ibuprofen | 600-800 mg 3 times daily | 1 to 2 weeks | Decrease by 200 mg every 1-2 weeks |
| Indomethacin | 25-50 mg 3 times daily | 1 to 2 weeks | Decrease by 25 mg every 1-2 weeks |
| Colchicine | - 0.5 mg twice daily - 0.5 mg once daily if body weight <70kg or severe renal impairment | 3 to 6 months | Not required, although suggested by some experts |
| Prednisone | 0.2-0.5 mg/kg/day | 2 to 4 weeks | Several months |

Readapted from: Schulz-Menger, J. et al., 2025 ESC Guidelines for the management of myocarditis and pericarditis. *European Heart Journal* (2025) vol. 46, 3952–4041; https://doi.org/10.1093/eurheartj/ehaf192.

**Supplementary Table 4. Clinical presentation of the overall cohort based on the occurrence of the 12-month composite outcome.**

|  | **Uncomplicated cases**  **(N=135)** | **Patients with composite outcome**  **(N=34)** | ***p*-value** |
| --- | --- | --- | --- |
| Pericardial chest pain, n (%) | 131 (97.0) | 32 (94.1) | 0.348 |
| Pericardial effusion, n (%) | 81 (62.8) | 26 (78.8) | 0.101 |
| Mild, n (%) | 60 (46.9) | 16 (50.0) | 0.844 |
| Moderate, n (%) | 16 (12.5) | 8 (25.0) | 0.096 |
| Severe, n (%) | 3 (2.3) | 1 (3.1) | >0.99 |
| Not measured, n (%) | 2 (1.5) | 1 (2.9) | 0.564 |
| ECG changes | 59 (48.9) | 19 (63.3) | 0.220 |
| Diffuse ST segment elevation, n (%) | 46 (34.1) | 13 (38.2) | 0.690 |
| PR segment depression, n (%) | 2 (1.5) | 2 (5.9) | 0.181 |
| Both, n (%) | 8 (5.9) | 5 (14.7) | 0.140 |
| Pericardial rubs, n (%) | 9 (6.7) | 2 (5.9) | >0.99 |

Legend. ECG: electrocardiogram.

**Supplementary Table 5. Predisposing factors for acute pericarditis based on the occurrence of the 12-month composite outcome.**

|  | **Uncomplicated cases**  **(N=135)** | **Patients with composite outcome**  **(N=34)** | ***p*-value** |
| --- | --- | --- | --- |
| Any viral infection in the past month, n (%) | 52 (38.5) | 13 (38.2) | >0.99 |
| Flu-like syndrome, n (%) | 39 (28.9) | 10 (30.3) | 0.952 |
| Gastroenteritis, n (%) | 15 (11.1) | 3 (9.1) | 0.699 |
| SARS-CoV-2 infection |  |  |  |
| Previous (any time), n (%) | 16 (11.9) | 6 (17.6) | 0.369 |
| Current, n (%) | –––– | 1 (2.9) | >0.99 |
| 1 week before, n (%) | –––– | 1 (2.9) | >0.99 |
| 1 month before, n (%) | –––– | 2 (5.9) | >0.99 |
| 2 to 6 months before, n (%) | 8 (5.9) | 2 (5.9) | 0.992 |
| 7 to 12 months before, n (%) | –––– | 3 (2.2) | >0.99 |
| >12 months before, n (%) | 1 (0.7) | –––– | >0.99 |
| SARS-CoV-2 vaccination |  |  |  |
| Type of vaccine |  |  |  |
| Comirnaty (Pfizer- BioNTech), n (%) | 33 (55.9) | 10 (47.6) | 0.553 |
| Spikevax (Moderna), n (%) | 24 (40.7) | 10 (47.6) | 0.130 |
| Vaxzevria (AstraZeneca), n (%) | 2 (3.4) | 1 (4.8) | 0.564 |
| ≥2 doses of any vaccine, n (%) | 59 (43.7) | 21 (61.8) | 0.083 |
| Time between vaccination and ED admission |  |  |  |
| 1 week, n (%) | 4 (3.0) | 1 (2.9) | 0.994 |
| 1 month, n (%) | 3 (2.2) | 4 (11.8) | 0.012 |
| 2 to 6 months, n (%) | 17 (12.6) | 5 (14.7) | 0.743 |
| 7 to 12 months, n (%) | 22 (16.3) | 5 (14.7) | 0.821 |
| >12 months, n (%) | 12 (8.9) | 3 (8.8) | 0.990 |
| Other vaccines in the previous month, n (%) | –––– | –––– | –––– |

Legend. ED: emergency department. PCI: percutaneous coronary intervention. PCP: percutaneous cardiac procedure. PM: pacemaker. SARS-CoV-2: severe acute respiratory syndrome coronavirus-2.

**Supplementary Table 6. Sensitivity analysis for the composite endpoint excluding major secondary causes.**

| **Variables** | **Full cohort (N=169)** |
| --- | --- |
| COPD | HR 6.21 (95% CI 2.26-17.09), p<0.001 |
| Type 2 diabetes | HR 3.93 (95% CI 1.71-9.07), p=0.001 |
| Recent PCPs | HR 3.01 (95% CI 1.10-8.25), p=0.032 |
| Recent SARS-CoV-2 vaccination | HR 6.49 (2.15-19.62), p<0.001 |
| Female sex | HR 1.96 (95% CI 0.98-3.91), p=0.056 |
|  |  |
| **Variables** | **Sensitivity cohort (N=151)** |
| COPD | HR 4.29 (0.98-18.88), p=0.054 |
| Baseline hemoglobin | HR 0.75 (95% CI 0.61-0.94), p=0.012 |
| Recent SARS-CoV-2 vaccination | HR 4.71 (95% CI 1.36-16.31), p=0.014 |

Legend. CI: confidence interval. COPD: chronic obstructive pulmonary disease. HR: hazard ratio. PCP: percutaneous cardiac procedure. SARS-CoV-2: severe acute respiratory syndrome coronavirus-2.

**Supplementary Table 7. Sensitivity analysis for the composite endpoint restricted to recurrences only.**

|  | **Univariable model**  **HR (95% CI)** | **p-value** |
| --- | --- | --- |
| Female sex | 2.64 (1.04-6.69) | 0.041 |
| Recent PCPs | 3.71 (1.07-12.86) | 0.039 |
| Baseline CRP, per mg/L | 1.01 (1.00-1.01) | 0.040 |
| Baseline ECG changes | 5.82 (1.30-26.03) | 0.021 |
| Baseline prednisone | 2.32 (0.53-10.15) | 0.263 |
| Baseline colchicine | 0.80 (0.30-2.17) | 0.666 |
| Baseline pericardial effusion | 1.94 (0.64-5.89) | 0.243 |
| Prior acute pericarditis | 2.23 (0.84-5.94) | 0.109 |
|  |  |  |
|  |  |  |
|  | **Multivariable model**  **HR (95% CI)** | **p-value** |
| **Model 1** |  |  |
| Baseline ECG changes | 5.24 (1.14 – 24.09) | 0.033 |
| Baseline CRP, per mg/L | 1.01 (1.00 – 1.02) | 0.006 |
| **Model 2** |  |  |
| Female sex | 2.97 (1.16 – 7.64) | 0.024 |
| Recent PCPs | 4.66 (1.32 – 16.44) | 0.017 |

Legend. CI: confidence interval. CRP: C-reactive protein. ECG: electrocardiogram. HR: hazard ratio. PCP: percutaneous cardiac procedure.

**Supplementary Table 8. Baseline characteristics of the overall cohort based on troponin T levels at the time of ED admission**

|  | **Troponin T <14 ng/L**  **(N=87)** | **Troponin T ≥14 ng/L**  **(N=75)** | ***p*-value** |
| --- | --- | --- | --- |
| **Demographics** |  |  |  |
| Age, years | 51 [39-59] | 62 [42-74] | 0.002 |
| Sex |  |  |  |
| Male, n (%) | 58 (66.7) | 45 (60.0) | 0.415 |
| Female, n (%) | 29 (33.3) | 30 (40.0) |  |
| **Vitals** |  |  |  |
| SBP, mmHg | 130 [116-140] | 128 [115-145] | 0.357 |
| DBP, mmHg | 80 [70-85] | 75 [67-85] | 0.184 |
| HR bpm | 89 [75-100] | 88 [75-104] | 0.876 |
| SpO_2_, % | 98 [97-99] | 97 [95-98] | 0.150 |
| Body temperature, °C | 36.4 [36.0-37.0] | 36.5 [36.0-37.0] | 0.923 |
| NRS scale | 5 [4-7] | 5 [2-8] | 0.963 |
| BMI, kg/m^2^ | 24.4 [21.8-29.0] | 25.4 [23.4-29.0] | 0.269 |
| **Medical history** |  |  |  |
| Hypertension, n (%) | 24 (27.6) | 40 (53.3) | 0.001 |
| Type 2 diabetes, n (%) | 4 (4.6) | 16 (21.3) | 0.002 |
| CAD, n (%) | 11 (12.6) | 17 (22.7) | 0.100 |
| Atrial fibrillation, n (%) | 7 (8.0) | 8 (10.8) | 0.595 |
| Chronic heart failure, n (%) | –––– | 9 (12.2) | <0.001 |
| CKD, n (%) | 2 (2.3) | 12 (16.0) | 0.003 |
| Obesity, n (%) | 11 (14.9) | 11 (16.2) | 0.707 |
| COPD, n (%) | 2 (2.3) | 5 (6.7) | 0.251 |
| Autoimmune disease, n (%) | 3 (3.4) | 7 (9.3) | 0.190 |
| Active cancer, n (%) | –––– | 2 (2.7) | 0.213 |
| Previous cancer, n (%) | 7 (8.0) | 6 (8.1) | >0.99 |
| Active smoking, n (%) | 26 (37.1) | 16 (23.9) | 0.100 |
| Prior radiotherapy, n (%) | 4 (4.7) | 1 (1.4) | 0.373 |
| Recent chest trauma, n (%) | –––– | –––– | –––– |
| Recent PCPs, n (%) | 3 (3.4) | 8 (10.7) | 0.068 |
| PCI and stenting, n (%) | 2 (2.3) | 6 (8.0) | 0.094 |
| PM implantation/cardiac ablation, n (%) | 1 (1.1) | 2 (2.7) | 0.475 |
| Recent cardiac surgery, n (%) | 1 (1.1) | 1 (1.3) | >0.99 |
| **Laboratory findings** |  |  |  |
| WBC, x10^9^/L | 10.5 [8.2-12.6] | 10.9 [8.4-13.0] | 0.442 |
| Hemoglobin, g/dL | 14.1 [12.8-15.2] | 13.4 [12.3-14.5] | 0.003 |
| Platelets, x10^9^/L | 261 [205-323] | 220 [193-290] | 0.043 |
| Creatinine, mg/dL | 0.9 [0.8-1.0] | 1.0 [0.8-1.3] | 0.002 |
| eGFR, mL/min/1.73 m^2^ | 94.5 [80.7-106.3] | 86.0 [60.0-102.0] | 0.002 |
| CRP, mg/L | 22.4 [3.1-57.2] | 58.4 [16.6-115.4] | <0.001 |
| Troponin T, ng/L | 5.0 [3.0-8.0] | 38.0 [20.0-111.0] | <0.001 |
| NT-proBNP, pg/mL | 182.0 [71.5-305.5] | 635.0 [177.0-1,883.0] | 0.006 |
| LDH, U/L | 177.5 [152.2-206.2] | 199.0 [167.2-234.8] | 0.003 |

Legend. ALT: alanine transaminase. AST: aspartate aminotransferase. BMI: body mass index. CAD: coronary artery disease. COPD: chronic obstructive pulmonary disease. CKD: chronic kidney disease. CRP: C-reactive protein. DBP: diastolic blood pressure. eGFR: estimated glomerular filtration rate. HR: heart rate. LDH: lactate dehydrogenase. NRS: numeric rating scale. NT-proBNP: N-terminal pro-B-type natriuretic peptide. PCI: percutaneous coronary intervention. SBP: systolic blood pressure. SpO2: saturation of peripheral oxygen. WBC: white blood cell.

**Supplementary Table 9. Clinical presentation of the overall cohort based on troponin T levels at the time of ED admission**

|  | **Troponin T**  **<14 ng/L**  **(N=87)** | **Troponin T**  **≥14 ng/L**  **(N=75)** | ***p*-value** |
| --- | --- | --- | --- |
| Pericardial chest pain, n (%) | 85 (97.7) | 72 (96.0) | 0.663 |
| Pericardial effusion, n (%) | 54 (65.1) | 48 (65.8) | >0.99 |
| Mild, n (%) | 42 (50.6) | 32 (45.1) | 0.521 |
| Moderate, n (%) | 10 (12.0) | 13 (18.3) | 0.365 |
| Severe, n (%) | 1 (1.2) | 1 (1.4) | >0.99 |
| Not measured, n (%) | 1 (1.2) | 2 (2.7) | 0.475 |
| ECG changes | 40 (51.3) | 35 (51.5) | >0.99 |
| Diffuse ST segment elevation, n (%) | 31 (35.6) | 27 (36.0) | >0.99 |
| PR segment depression, n (%) | 4 (4.6) | –––– | 0.124 |
| Both, n (%) | 6 (6.9) | 6 (8.0) | 0.789 |
| Pericardial rubs, n (%) | 8 (9.2) | 3 (4.0) | 0.225 |

Legend. ECG: electrocardiogram.

**Supplementary Table 10. Predisposing factors for acute pericarditis based on troponin T levels at the time of ED admission.**

|  | **Troponin T**  **<14 ng/L**  **(N=87)** | **Troponin T**  **≥14 ng/L**  **(N=75)** | ***p*-value** |
| --- | --- | --- | --- |
| Any viral infection in the past month, n (%) | 33 (37.9) | 30 (40.0) | 0.872 |
| Flu-like syndrome, n (%) | 25 (29.1) | 22 (29.3) | >0.99 |
| Gastroenteritis, n (%) | 9 (10.5) | 9 (12.0) | 0.806 |
| SARS-CoV-2 infection |  |  |  |
| Previous (any time), n (%) | 14 (16.1) | 8 (10.7) |  |
| Current, n (%) | 1 (1.1) | –––– |  |
| 1 week before, n (%) | 1 (1.1) | –––– |  |
| 1 month before, n (%) | 1 (1.1) | 1 (1.3) |  |
| 2 to 6 months before, n (%) | 7 (8.0) | 3 (4.0) |  |
| 7 to 12 months before, n (%) | 3 (3.4) | –––– |  |
| >12 months before, n (%) | 1 (1.1) | –––– |  |
| SARS-CoV-2 vaccination |  |  |  |
| Type of vaccine |  |  |  |
| Comirnaty (Pfizer- BioNTech), n (%) | 20 (23.0) | 22 (29.3) | 0.358 |
| Spikevax (Moderna), n (%) | 15 (17.2) | 19 (25.3) | 0.207 |
| Vaxzevria (AstraZeneca), n (%) | 2 (2.3) | –––– | 0.500 |
| ≥2 doses of any vaccine, n (%) | 37 (42.5) | 42 (56) | 0.115 |
| Time between vaccination and ED admission |  |  |  |
| 1 week, n (%) | 1 (1.1) | 4 (5.3) | 0.117 |
| 1 month, n (%) | 5 (5.7) | 2 (2.7) | 0.336 |
| 2 to 6 months, n (%) | 10 (11.5) | 13 (17.3) | 0.288 |
| 7 to 12 months, n (%) | 15 (17.2) | 12 (16.0) | 0.832 |
| >12 months, n (%) | 5 (5.7) | –––– |  |
| Other vaccines in the previous month, n (%) | –––– | –––– | –––– |

Legend. ED: emergency department. PCI: percutaneous coronary intervention. PCP: percutaneous cardiac procedure. PM: pacemaker. SARS-CoV-2: severe acute respiratory syndrome coronavirus-2.

**Supplementary Figure 1. Participant flow of the OSAP-VED study**

**Supplementary Figure 2. Characteristics of presentation to the Emergency department.**

**A**: Season of the year. **B**: Setting of origin. **C**: Way of presentation at the Emergency department. **D**: Color-coded categories assigned at the Emergency department triage.

**Supplementary Figure 3. Prescription of prednisone and doses.**

**Panel A**. Proportion of patients on prednisone at admission and discharge. **Panel B**. Mean daily dose of prednisone at admission and discharge. * p<0.05 for McNemar’s test; *** p<0.0001 for paired sample Student’s t-test.

**Supplementary Figure 4. Proportion of the primary endpoint according to guideline-recommended drug doses at discharge.**

Proportion of patients experiencing the primary outcome according to guideline-recommended drug doses at discharge. p for chi-squared test.
